# From Excellent Clinicians to Custodians of Life: Reimagining Internationalization in Health Professions Education

**DOI:** 10.5334/aogh.5146

**Published:** 2026-04-13

**Authors:** Lilian Ferrer, Felipe Heusser, Javiera Fuentes, Javier Kattan, Camila Lucchini, Patricio Smith, Andrea Moreno, Claudia Bambs

**Affiliations:** 1 School of Nursing, Faculty of Medicine, Pontificia Universidad Católica de Chile, Santiago, Chile; 2 Faculty of Medicine, Pontificia Universidad Catolica de Chile, Santiago, Chile; 3 School of Health Allies, Faculty of Medicine, Pontificia Universidad Católica de Chile, Santiago, Chile; 4 School of Medicine, Faculty of Medicine, Pontificia Universidad Católica de Chile, Santiago, Chile; 5 School of Dentistry, Faculty of Medicine, Pontificia Universidad Católica de Chile, Santiago, Chile; 6 School of Veterinary Medicine, Faculty of Medicine, Pontificia Universidad Católica de Chile, Santiago, Chile; 7 School of Public Health, Faculty of Medicine, Pontificia Universidad Católica de Chile, Santiago, Chile

**Keywords:** global competences, health care professional, equity and sustainability in health education

## Abstract

Health professions education faces growing challenges such as climate disruption, migration, inequities, and information-driven mistrust. Preparing health professionals for this reality requires more than clinical excellence; it calls for reimagining internationalization as the intentional integration of global, intercultural, ethical, interprofessional, and ecological competencies across curricula and accreditation systems. This commentary argues that internationalization must move beyond mobility for a privileged few toward a universal framework of global competencies accessible to all students. Drawing on frameworks from UNESCO, World Health Organization, and the Lancet Commission, and illustrated through the experience of the Faculty of Medicine at Pontificia Universidad Católica de Chile, it outlines three strategic directions: reorienting accreditation, embedding global engagement into curricula, and fostering authentic interdisciplinary teamwork. Ultimately, this paper offers a normative framework and institutional illustration to guide faculties of health worldwide in preparing graduates as custodians of life in a shared and fragile world.

## Introduction

Universities often celebrate their ability to produce excellent professionals. Yet the crises shaping this century, including climate disruption, forced displacement, growing inequities, and the erosion of trust in science and politics, demand something far greater. If internationalization in higher education cannot prepare health professionals to act as custodians of life and democracy in an interconnected and fragile world, then its purpose deserves urgent re-examination.

In this commentary, internationalization is understood not as mobility alone but as the deliberate integration of global, intercultural, ethical, and interprofessional learning into the core of health professions education. Rather than an isolated activity, it is framed here as a way of strengthening the social responsibility of universities and their contribution to more just, sustainable, and equitable health systems. This approach aligns with the ethical vision of James Keenan [1], suggesting that internationalization must be reclaimed as a tool for the “Common Good,” shifting the focus from institutional prestige to an ethical responsibility toward the most vulnerable in our global community.

As UNESCO [2, 3] has stated, the mission of higher education extends far beyond the transmission of knowledge. It must also cultivate graduates capable of contributing to peaceful, just, and sustainable societies. Similarly, the World Health Organization [4, 5] has emphasized interprofessional and socially accountable education, while the *Lancet* Commission on the Education of Health Professionals called for a “third generation” of reform that is systems-based, competency-driven, and globally connected [6]. These perspectives align with the Rockefeller Foundation–Lancet Commission on Planetary Health, which highlights that protecting human health depends on safeguarding the natural systems that sustain life [7].

Taken together, these frameworks suggest that internationalization, when reimagined, can become one of the most powerful vehicles for aligning health professions education with the public good, including the impact of globalization on democratic norms and the principles of human rights that support them.

## Rethinking Global Competencies: Three Strategic Directions

The *Lancet* Commission [6] called for a third generation of reform. For internationalization in health professions education to move beyond mobility and prestige, it must be anchored in deliberate shifts that reorient entire systems of learning. We suggest three deeply interconnected directions. They are not technical adjustments but rather normative choices—informed by a commitment to the public good [8]—that redefine what higher education values, measures, and rewards.

### 1. Accreditation that values global competencies and social accountability

Accreditation and rankings are powerful signals. They shape what faculties measure, improve, and invest in. Yet most quality assurance systems still privilege tangible inputs and outputs such as infrastructure, research metrics, or licensing exam scores over the outcomes that truly matter in a globalized world. Competencies such as intercultural humility, systems thinking, interprofessional teamwork, and social accountability remain largely invisible in accreditation frameworks.

This invisibility has consequences. When accreditation does not require evidence of global or intercultural learning, faculties often underinvest in initiatives that build precisely those skills. To prepare graduates for complex realities, accrediting bodies must ask different questions: Do students demonstrate the ability to work across cultures and disciplines? Do programs align with the health needs of both local and global communities? Do institutions document their contributions to equity, sustainability, social cohesion, and protection of democracy?

These questions are uncomfortable because they challenge universities to look beyond traditional prestige indicators. Yet unless accreditation evolves, internationalization will remain peripheral, celebrated in glossy brochures but absent from the daily curriculum. As we will discuss in our Case Illustration, addressing these challenges requires structural changes, such as the integration of a permanent Quality Assurance area to ensure that these global competencies are not just aspirational, but measurable and sustained.

### 2. International engagement as a right, not a privilege

The dominant model of internationalization, focused on sending a few students abroad, excludes the majority. Financial barriers, visa restrictions, language limitations, and caregiving responsibilities prevent many from participating in such opportunities. In health professions education, this exclusion is particularly problematic, as those who cannot travel are often the very graduates who will later serve under-resourced communities.

A new vision is needed, one that ensures every student has access to meaningful, curriculum-integrated global learning. This can take many forms: Collaborative Online International Learning (COIL), local-global projects with migrant or Indigenous communities, short and structured mobility programs, or language-across-the-curriculum initiatives. These approaches are not supplementary experiences but central elements that can be credit-bearing, embedded in core courses, and assessed for intercultural communication, ethical collaboration, and systems thinking.

The challenge lies in thoughtful design. Poorly planned “international projects” risk being tokenistic or even extractive. However, intentional, scaffolded, and ethically grounded engagement—rooted in what James Keenan describes as the ethical responsibility toward the “Common Good”—can generate transformative learning at relatively low cost. The key principle is equity: internationalization must serve the entire student body, not only the privileged few.

### 3. Interdisciplinary and interprofessional learning through authentic problems

The challenges facing health professionals—antimicrobial resistance, climate-related diseases, mental health in displaced populations, and the impact of polarized and fragile sociopolitical contexts—cannot be solved by any single discipline. Preparing students to face such complexity requires interprofessional, interdisciplinary, and intercultural collaboration to become the pedagogy itself, rather than an optional activity.

Authenticity is essential. Students learn collaboration best when they engage with real problems co-constructed with communities, such as rural clinics, primary care networks, public health initiatives, and rehabilitation services. These experiences expose them to the tensions and trade-offs of practice, including scarce resources, cultural diversity, and ethical dilemmas. In such contexts, learners must negotiate roles, integrate perspectives, and develop trust across boundaries.

This kind of education also demands courage from institutions. It requires faculty to share authority with communities, to assess not only technical knowledge but also teamwork and intercultural competence, and to recognize that the most valuable learning often happens outside lecture halls. Without such experiences, graduates risk entering practice unprepared for the collaborative realities of global health care.

Each of these three directions—reimagined accreditation, equitable access to global engagement, and authentic interprofessional learning—leads to the same conclusion: internationalization must be redefined as a shared responsibility. It is not simply about producing graduates who can navigate airports or conferences, but about preparing professionals who can navigate the ethical, ecological, and social complexities of a fragile planet.

What is at stake is far more than curriculum design. It is a redefinition of what success means in higher education. Do we judge faculties by the number of indexed publications, or by their graduates’ capacity to serve diverse populations ethically and sustainably? These questions reveal the deeper values that internationalization either reinforces or challenges. The answers will not be the same everywhere, yet the direction is clear: health professions education must cultivate **custodians of life**—graduates who combine clinical skill with global responsibility.

## Case Illustration: UC Chile’s Faculty of Medicine

The argument for embedding global competencies in health professions education can seem abstract until we see how institutions are putting it into practice. One example comes from the Faculty of Medicine at Pontificia Universidad Católica de Chile (UC Chile).

The Faculty comprises five core schools—Medicine, Nursing, Nutrition, Dentistry, and the School of Health Sciences (which integrates Physiotherapy, Occupational Therapy, and Speech Pathology)—and the recently established School of Public Health. A distinctive feature of this ecosystem is the **School of Veterinary Medicine**, which functions as an **Associate School**. This unique entity operates under a shared governance model between the Faculty of Medicine, the Faculty of Biological Sciences, and the Faculty of Agronomy and Natural Resources. This multi-professional environment creates a “One Health” laboratory where collaboration across human, animal, and environmental disciplines is both possible and necessary.

In recent years, the faculty made a strategic decision to integrate internationalization into the core of its institutional structure. The creation of the **Direction of Global Affairs** marked a turning point. Its mandate is to align curricula, partnerships, and evaluation mechanisms across all schools. Rather than focusing solely on prestige-based agreements, this unit embeds competencies such as intercultural communication and ethical global engagement into every program.

In practice, this shift has taken several forms:

- Curricular Mapping: Programs are developing shared rubrics to evaluate teamwork and intercultural reflection alongside clinical skills.
- Diversified Engagement: Beyond traditional mobility, students participate in COIL modules and community-based projects with migrant and Indigenous populations in Chile. To support this, the Faculty is currently in the process of accommodating resources to secure international mobility for at least 10% of the students within each program.
- Internationalization at Home: The Faculty has prioritized the active reception of international students, establishing structured ways for local and visiting peers to interact. This is designed to develop the interculturality and flexibility graduates need to care for people from different backgrounds.
- Authentic Collaboration: Interprofessional initiatives bring together students from all five schools and the Associate School of Veterinary Medicine. These have included collaborations in Indonesia and Africa, supported by diplomatic partners to ensure trust and reciprocity.
- Quality Assurance: In 2025, a new Quality Assurance and Continuous Improvement Unit was created. This unit ensures that global competencies and social accountability are permanently enhanced and monitored, rather than being addressed only during accreditation cycles.

The UC Chile case illustrates that when universities in the Global South align accreditation, curriculum, and pedagogy around global competencies, they send a powerful message: internationalization is not the preserve of elite institutions, but a shared responsibility. By reframing internationalization as part of the institutional mission to serve society, we can educate graduates who are not only excellent clinicians but also true custodians of life.

## Call to Action: Toward Custodians of Life

Reimagining internationalization in health professions education is not simply a curricular reform. It is a question about what kind of professionals universities wish to form and what kind of societies they aspire to serve. At stake is nothing less than the alignment between higher education and the global public good.

The three shifts outlined—reframing accreditation, embedding international engagement into curricula, and fostering authentic interdisciplinary learning—are neither simple nor universally accepted. They challenge long-standing traditions in higher education that reward prestige, competition, and individual achievement. They require institutions to recognize and assess what has often remained invisible: intercultural humility, ethical collaboration, reciprocity with communities, and commitment to sustainability, democracy, and human rights. These outcomes are more difficult to measure than test scores or publication counts, yet they are far more consequential for our shared future. This shift represents an ethical move toward what James Keenan describes as the “Common Good,” where the success of an institution is measured by its contribution to the most vulnerable.

Several barriers make this transformation challenging. Resource limitations often lead faculties to argue that internationalization is unaffordable. National accreditation agencies may be slow to include global competencies in their standards. Faculty members may resist new pedagogies, especially those requiring co-teaching, cross-border collaboration, or community engagement. Communities themselves may also be hesitant, particularly if their previous experiences with universities left them feeling used rather than valued.

These barriers are real, but they are not insurmountable. In fact, they highlight why a deeper shift in values is essential. Internationalization does not have to depend on vast resources; it can begin with low-cost, high-impact initiatives such as COIL modules or community-based, co-constructed projects. Accreditation inertia can be addressed through pilot experiences that demonstrate educational and social value. Faculty resistance can be reduced through incentives, recognition, and professional development. Community mistrust can be addressed through reciprocity, transparency, and sustained partnerships. What initially appears to be a list of obstacles can, with intention and creativity, become a set of catalysts for innovation.

Although this commentary focuses on health professions education, its implications extend across higher education. Engineering, law, business, and the arts face similar challenges: how to prepare graduates for a world marked by ecological fragility, technological disruption, geopolitical tensions, and deepening inequities. The call to embed global competencies, ensure equitable access to international learning, and promote authentic collaboration resonates far beyond the health sector. Health, however, offers an urgent and visible test case, because when education fails to prepare professionals for global complexity, the consequences are measured not only in inefficiencies but in human lives.

While many of these ideas have been widely discussed in global policy and academic literature, they have been less frequently examined from the perspective of Latin American and other Global South contexts. These settings offer critical insights into equity, resource constraints, social accountability, and community engagement. They also demonstrate that meaningful internationalization can emerge even in environments with limited resources, if it is guided by intentionality, reciprocity, and a commitment to the public good [8].

Ultimately, the metaphor of “custodians of life” is not rhetorical. It reminds us that health professionals, and indeed all graduates, carry responsibilities that transcend their disciplines. They are entrusted with the well-being of communities, ecosystems, and future generations. Preparing them for this role requires cultivating capacities that go beyond clinical or technical knowledge: empathy across cultures, humility in collaboration, courage in ethical dilemmas, and imagination for sustainable futures.

Internationalization, when redefined through the lens of global competencies, becomes one of the most powerful instruments higher education possesses to meet these responsibilities. Yet this potential will only be realized if internationalization is universal, intentional, and accountable. Otherwise, it risks remaining an exclusionary practice that reinforces privilege rather than transforming societies.

## Conclusion: An Invitation to Global Health Dialogue

This commentary does not seek to present a definitive model. Rather, it offers an invitation to dialogue—a call to rethink how internationalization can better serve equity, ethics, and sustainability in health professions education. The experience of the Faculty of Medicine at UC Chile represents only one possible pathway. The real task ahead is collective: to adapt, contextualize, and co-create approaches that reflect diverse realities and shared responsibilities in advancing global health, particularly in settings marked by inequality and resource constraints.

This also requires examining how internationalization is currently practiced within health professions education. Accreditation systems must move beyond technical and disciplinary competencies to incorporate global responsibility, intercultural understanding, and social accountability. Curricula should ensure that every student—not only those who can travel—has access to meaningful opportunities to develop global competencies. Equally important, institutions must engage communities as genuine partners in knowledge co-creation rather than distant beneficiaries of academic initiatives.

The goal is not to replicate a single institutional model, but to nurture generations of graduates prepared to act as custodians of life—professionals capable of navigating complexity, working across disciplines and cultures, and responding ethically to the interconnected challenges of climate change, migration, inequities, and fragile health systems.

Ultimately, the question facing faculties of health worldwide is both simple and profound: will institutions continue to reward prestige and privilege, or will they reorient themselves toward the public good and the health of populations? The answer will shape not only the future of health professions education, but also the well-being of the communities and ecosystems on which health depends. By redefining internationalization as a shared responsibility, faculties of health can move beyond prestige and competition to contribute more meaningfully to global health, sustainability, and social justice [8, 9].
